# Biogenic Synthesis and Spatial Distribution of Endogenous Phytohormones and Ginsenosides Provide Insights on Their Intrinsic Relevance in *Panax ginseng*

**DOI:** 10.3389/fpls.2018.01951

**Published:** 2019-01-09

**Authors:** Kang Chen, Juan Liu, Ruifeng Ji, Tong Chen, Xiuteng Zhou, Jian Yang, Yuru Tong, Chao Jiang, Junhui Zhou, Yuyang Zhao, Yan Jin, Yuan Yuan, Luqi Huang

**Affiliations:** ^1^Anhui University of Chinese Medicine, Hefei, China; ^2^National Resource Center for Chinese Materia Medica, Chinese Academy of Chinese Medical Sciences, Beijing, China

**Keywords:** *Panax ginseng*, phytohormone, jasmonic acid, salicylic acid, abscisic acid, gibberellins, ginsenoside biosynthesis

## Abstract

Ginseng, the root and rhizome of *Panax ginseng* C. A. Mey., is a well-known and valuable traditional Chinese medicine. The pharmacological activities of ginseng are mainly attributed to the presence of ginsenosides, which are considered to be critical biomarkers for evaluating ginseng quality. The biosynthesis of triterpenes, which serve defensive functions in plants, is regulated by endogenous phytohormones that play key roles in growth and defense of plant populations. However, the role of major hormones that are closely related to secondary metabolism pathways in *P. ginseng* is poorly understood. To gain insight into their potential correlation, we performed a spatial synthesis analysis and studied the distribution of endogenous phytohormones and ginsenosides in different tissue regions of the entire *P. ginseng* plant. Gibberellins are growth hormones that accumulate in the fiber root. In contrast, abscisic acid (ABA), salicylic acid (SA), and jasmonic acid (JA), which are considered stress hormones, were predominantly found in the leaf and leaf peduncle. We observed a tissue-specific distribution of phytohormones consistent with the expression of genes involved in hormone biosynthesis that influenced ginsenoside synthesis and distribution. The aim of this study was to investigate the role of different endogenous phytohormones on triterpene metabolites in ginseng innate immunity.

## Introduction

**Figure 1:**
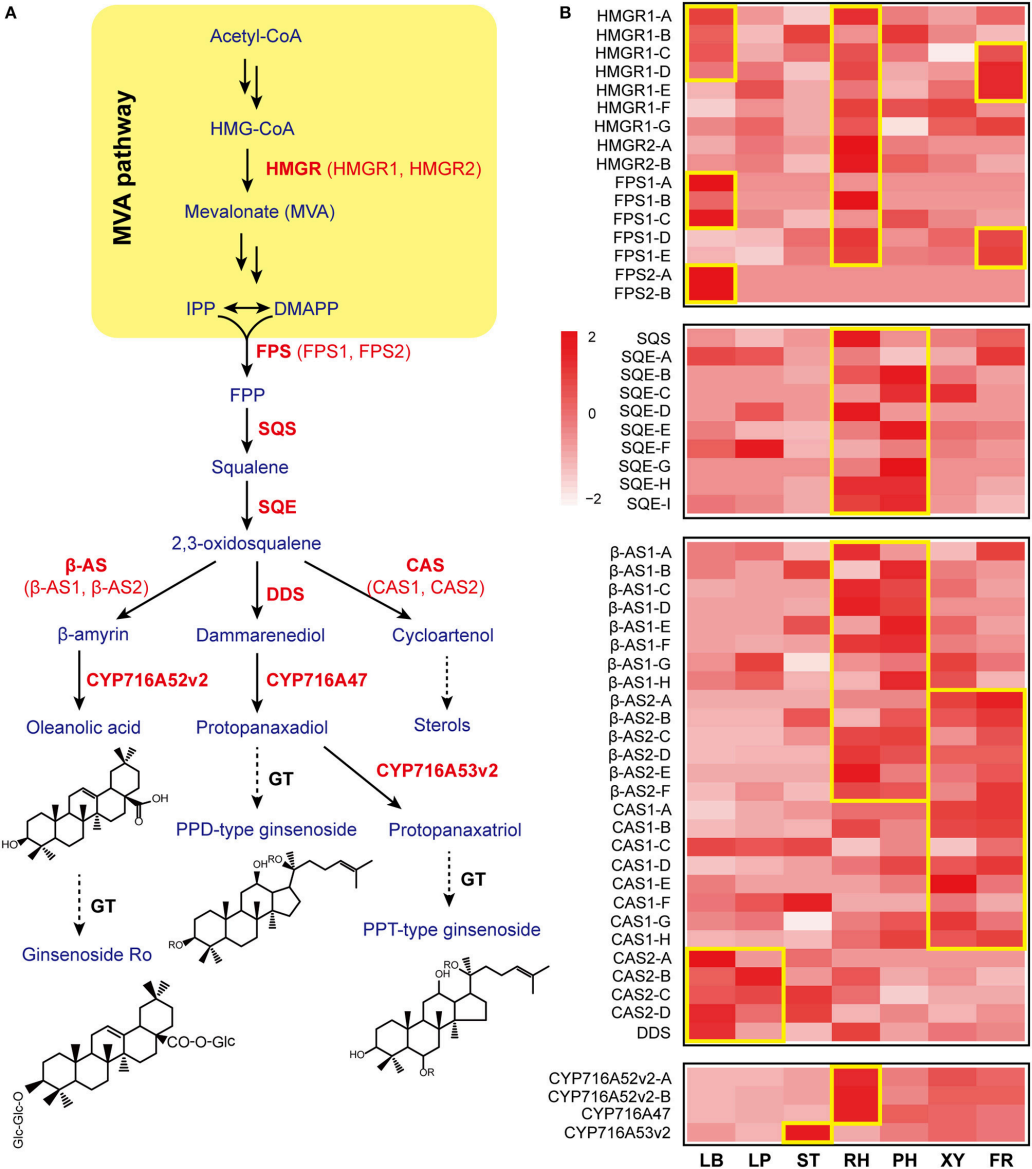
Gene expression patterns involved in ginsenoside biosynthesis. **(A)** The ginsenoside biosynthetic pathway is shown. β-AS, β-amyrin synthase; CAS, cycloartenol synthase; CYP, cytochrome P450 proteins; DDS, dammarenediol-II synthase; FPP, farnesyl diphosphate; FPS, farnesyl diphosphate synthase; HMGR, 3-hydroxy-3-methylglutaryl-CoA reductase; SQE, squalene epoxidase; SQS, squalene synthase. The key enzymatic steps analyzed in this study are highlighted in red. **(B)** A heatmap of gene expression related to ginsenoside biosynthesis is shown. Each column represents one tissue (LB, leaf blade; LP, leaf peduncle; ST, stem; RH, rhizome; XY, xylem in the main root; PH, phloem and periderm in the main root; FR, fibrous root) and each row represents one unigene corresponding in RNA-seq data (Supplementary Table S1). Different unigene labels might match to one annotated gene here due to the same specific sequence regions or domains. Colors show Z-score transformed gene expression values among all samples. The units indicate the expression levels of key genes involved in ginsenoside biosynthesis in different tissues (only shown where the gene expression level RPKM>1). Red indicates an increase in expression, and white indicates a decrease in expression; color intensity indicates the magnitude of the effect.

Asian ginseng (*Panax ginseng* C.A. Meyer) has been recognized as a key medicinal herb and has played an indispensable role in the culture of traditional Chinese medicine for thousands of years in Eastern Asia (Hemmerly, 1977). As a symbolic herb of traditional Chinese medicine, ginseng has historically been ascribed as a general tonic to maintain the body's balance and resistance to adverse factors. Among the components in ginseng, ginsenosides have been shown to be a major pharmacological ingredient that are also major secondary metabolites in ginseng. To date, more than 110 natural ginsenosides have been isolated from *P. ginseng* and have been classified as either a dammarane type (e.g., protopanaxadiol [PPD], protopanaxatriol [PPT], and ocotillol) or oleanane type (Qi et al., 2011). The ginsenoside biosynthetic pathway has been largely elucidated (Kim et al., 2015) (Figure 1A). Chemical analysis and histochemical staining have shown that ginsenosides frequently accumulate in specific tissues, especially in the oil canals of the periderm and outer cortex regions of the root, suggesting that phloem and resin ducts are metabolically active sites for both sterol and ginsenoside biosynthesis (Attele et al., 1999). However, the mechanisms underlying the biosynthesis of a variety of ginsenosides in specific tissues and their transport to target tissues in *P. ginseng* have yet to be elucidated. Interestingly, it has been suggested that some phytohormones contribute to the regulation of ginsenoside biosynthesis (Yu et al., 2002).

Phytohormones, as a group of naturally organic substances, play crucial roles in various plant physiological processes. To date, nine phytohormone families have been identified in plants that have characteristic biological functions, including auxins, jasmonic acids (JA) (Xu et al., 2002), gibberellins (GA) (Yamaguchi, 2008), salicylic acid (SA), abscisic acid (ABA) (Shen et al., 2006), ethylene, cytokinins, strigolactones (Al-Babili and Bouwmeester, 2015), and brassinosteroids. Increasing evidence shows that phytohormone concentration and distribution are determinants of phytohormone action (Kudo et al., 2013). Moreover, a vast amount of information has been obtained detailing the role of different phytohormones on plant secondary metabolites (Mithöfer and Boland, 2012). Jasmonic acid is a crucial signal transducer during wound stress or fungal-induced secondary metabolite formation in plants, such as carrots (Wang et al., 2016), tomatoes (Chen et al., 2006), *Nicotiana attenuata* (Ferrieri et al., 2015), and *Catharanthus roseus* (Peebles et al., 2009). Reactive oxygen species are vital for mediating plant immunity and secondary metabolism, which are closely related to accumulation of SA (Herrera-Vasquez et al., 2015). In addition, accumulation of SA induces the production of phenolic compounds, anthraquinones, and terpenes by increasing the expression amount of secondary metabolic genes or raising the activity of secondary metabolic enzymes (Bulgakov et al., 2002; Dong et al., 2010; Liu et al., 2015). Researchers have investigated that exogenous jasmonates (JAs) and salicylic acid (SA) to determine if they enhance the accumulation of ginsenosides in *P. ginseng* roots (Yu et al., 2002). Similarly, the influence of ABA and GAs on secondary metabolism in plants has been shown in *Arabidopsis thaliana* (Rock and Zeevaart, 1991) and grapes (Villalobos-González et al., 2016). Abscisic acid (ABA) accelerates sugar and anthocyanin accumulation to enhance the transcription of genes involved in pigment metabolism (Mou et al., 2015), and GA regulates the accumulation of alkaloids and gene expression of terpene synthases in plants (Schmiderer et al., 2010).

Although recent studies have shown that exogenous phytohormones enhance the accumulation of ginsenosides, no efforts have been made toward understanding the in-depth biogenic synthesis and spatial distribution of endogenous phytohormones in combination with the dynamics of ginsenoside accumulation and biosynthesis from various tissues. The therapeutic applications of *P. ginseng* are diverse; therefore, it is of great interest to perform spatial synthesis and distribution of endogenous phytohormone analyses to better understand the metabolic content and molecular basis of ginsenoside biosynthesis. In this manuscript, we present the biogenic synthesis and spatial distribution of four hormones (JA, SA, ABA, and GA_3_) in seven different tissues (leaves, petioles, stems, rhizomes, phloem and periderm in the main root, xylem in the main root, and fibrous roots) of *P. ginseng* to understand the indispensable relationship between endogenous phytohormones and ginsenoside biosynthesis in *P. ginseng*. Furthermore, these data shed light on the actual interactions between the level of plant hormones and the accumulation of triterpene metabolites *in vivo*. Understanding the significant role of endogenous phytohormones in ginsenoside biosynthesis will pave the way for large-scale production of these highly valued compounds using metabolic engineering tools and large-scale production of ginsenosides via plant-root cultures.

## Materials and Methods

### Plant Materials and Sample Collection

Three-year-old ginseng seedlings were mainly supplied by the Kangmei Pharmaceutical Company planting base and were identified as *P. ginseng* C.A. Meyer by Prof. Luqi Huang (China Academy of Chinese Medical Sciences). The materials (five strains of ginseng) were collected and divided into seven parts (leaves, petioles, stems, rhizomes, phloem and periderm in the main root, xylem in the main root, and fibrous roots) (LB, leaf blade; LP, leaf peduncle; ST, stem; RH, rhizome; XY, xylem in the main root; PH, phloem and periderm in the main root; FR, fibrous root). The samples were stored at −80°C and deposited at the National Resource Center for Chinese Materia Medica, China Academy of Chinese Medical Sciences, Beijing, China.

### RNA-Seq Library Splicing and Annotation

To detect and characterize the expressed mRNAs in different parts of *P. ginseng*, the raw sequences of seven RNA-seq libraries (leaf blade, leaf peduncle, stem, rhizome, phloem and periderm in the main root, xylem in the main root, and fiber root) were downloaded from the National Center for Biotechnology Information (NCBI) Sequence Read Archive (SRA: PRJNA302556). These sequenced reads were filtered to remove adaptors and low-quality bases. We then used Trinity (v2.2.4) to construct reference transcriptomes. Assembled unigenes were annotated against the NCBI Nr database, Uniprot Swiss-prot database, and Pfam database using Trinotate at a cutoff *E*-value < 1e-05. Gene ontology and Kyoto Encyclopedia of Genes and Genomes (KEGG) annotations were extracted from the Trinotate results. All gene information involved in ginsenoside biosynthesis and hormone metabolism is listed in Supplementary Tables S1, S2. Each gene label represents one unigene assembled using Trinity (Supplementary Tables S1, S2). These genes were searched in Uniprot (Swiss-Prot and TrEMBL) database to find the closest match for predicting the potential functions of assembled genes. Different unigene labels may match to one annotated gene for their specific sequence regions or domains. The labeled letter in Figure 1B and Supplementary Table S1 was corresponding with each other, the same as Figures 4B–E and Supplementary Table S2.

### Gene Expression Profiles

Gene expression values for genes involved in the biosynthetic pathways of ginsenosides and four phytohormones (ABA, JA, SA, and GA) were generated using RSEM software and are represented as the number of transcripts per million transcripts (TPM). Gene expression raw counts were fed to the R package edgeR for cross-sample normalization and differential expression gene identification. Differentially expressed genes were screened with absolute fold changes no <2 and multiple test corrected *p* values <0.05. The gene expression heat-maps of the ginsenoside biosynthesis in Figure 1B and hormone metabolism in Figures 4B–E were showed the expression of the corresponding unigenes in RNA-seq data in Supplementary Tables S1, S2, respectively.

### RNA Isolation, Transcriptome Sequencing and Real-Time PCR Analysis

Total RNA was isolated from the different tissues of *P. ginseng* using the TRIzol reagent (Invitrogen) from three biological replicates. Template cDNAs were prepared using PrimeScriptTM RT reagent Kit (Takara) from 2.0 μg total RNA following the manufacturer's instructions.

The unigenes used for the primers designed in qPCR were labeled in red (Supplementary Tables S1, S2). All gene specific primers for real-time PCR analysis were designed using Primer Premier (V5.0) software, as listed in Supplementary Table S3. All gene sequences involved in real-time PCR were listed in Supplementary Table S4. The *IF3G1* gene from *P. ginseng* was selected as the internal reference gene (Wang and Lu, 2015; Liu et al., 2017). These reactions were performed on a Roche LightCycler 480 Real Time PCR System, with the reaction mixture (20 μL) containing 10 μL 2 × Master Mix from KAPA SYBR® FAST qPCR kits (KAPABIOSYSTEMS), 10 μmol·L^−1^ forward and reverse primers (0.5 μL each), and 1 μL template cDNA. The PCR program in this case was 95°C for 3 min, followed by 50 cycles at 95°C for 3 s and 60°C for 30 s. Three independent biological replicates were performed for each sample, while the relative expression levels of the selected unigenes were calculated using the relative 2-ΔCT method (Livak and Schmittgen, 2001). The results represented in this case represent mean standard deviations for the three experimental replicates.

### Instruments, Chemicals, and Reagents

The analysis system included an Absciex 6500QTRAP three triple quadrupole mass spectrometer; ultrapure water system, Millipore, ThermoScientific, USA; BT-224S 1/10,000 electronic analytical balance; THZ-C-1 constant temperature shaker; and 5810R high speed refrigerated centrifuge. All standards were purchased at Sigma, including the ABA control (CAS:14375-45-2); JA (CAS:77026-92-7), methyl-JA (CAS:39924-52-2), JA-isoleucine (JA-Ile, LAR-13-1208-1), GA_3_ (CAS:77-06-5), SA (CAS:69-72-7), ^2^H_6_-ABA (CAS: 35671-08-0), ^2^H_2_-JA (CAS: 903510-50-9), ^2^H_4_-SA (CAS:78646-17-0), and ^2^H_2_-GA_4_ (CAS: 84693-21-0). All ginsenosides were purchased from Shanghai Winherb Medical Science Co. Ltd., including Re (CAS:51542-56-4), Rg1 (CAS:22427-39-0), Rb1 (CAS:41753-43-9), Rf (CAS:52286-58-5), Rd (CAS:52705-93-8), and Rb3 (CAS:68406-26-8). Purified acetonitrile and formic acid were purchased from Fisher. Analytic grade methanol, ethanol, 2-propanol, and dichloromethane were obtained from Sinopharm Chemical Reagent Co., Ltd. (Shanghai, China). The ultrapure water was produced in the authors' laboratory.

## Extraction of Phytohormones

The sample treatment method is based on previous research (Pan et al., 2010). The seven parts of the ginseng plant were frozen while still fresh using liquid nitrogen in mortar; 0.1 g of each sample was accurately weighed and placed into a 5-mL centrifuge tube. Throughout the whole process, the samples were maintained in liquid nitrogen. The extraction solvent (1 mL, 2-propanol/H_2_O/concentrated HCl [2:1:0.002, vol/vol/vol]) was added to each tube. If more than 0.1 g fresh starting plant tissue was used, the solvent volume was adjusted by keeping the ratio of sample to solvent at 1:10 (mg·μl^−1^). The tubes were then placed on a shaker at a speed of 100 rpm for 30 min at 4°C. Dichloromethane (1 mL) was added to each sample; the tubes were placed on the shaker for an additional 30 min at 4°C and a speed of 100 rpm and then centrifuged for 5 min at 13,000 × g and 4°C. After centrifugation, two phases were formed, with the plant debris located between the two layers. Solvent from the lower phase (1,800 μL) was then transferred into a new tube and concentrated, but not completely dried, using a nitrogen generator with nitrogen flow. Finally, the concentrated samples (10 μL) were dissolved in 50% methanol (400 μL) and prepared for high-performance liquid chromatography electrospray ionization tandem mass spectrometry (HPLC–ESI–MS/MS) analysis.

### Extraction of Ginsenosides

The ginseng samples were treated similarly as mentioned above. The seven parts of the ginseng plant were frozen while still fresh using liquid nitrogen in mortar; 0.1 g of each sample was accurately weighed and placed into a 5-mL centrifuge tube. Ethanol (2 mL, 70%) was added to each tube as an extraction solvent, which was then ultrasonic extracted for 30 min at 25°C and 25 KHz (SCIENT ultrasonic processor, Ningbo, China). After being centrifuged at 13,000 rpm for 10 min, the sample supernatants were subsequently filtered through a 0.2 μm membrane filter prior to analysis.

### Chromatography Mass Spectrometry Conditions

For phytohormone determinations, we used a Waters ACQUITYUPLCBEH C18 column (2.1 mm × 100 mm, 1.7 μm), with a mobile phase consisting of a 0.05% formic acid and water—acetonitrile solution. Linear elution gradients were 0~0.3 min, 10% acetonitrile; 0.3~3 min, 10~60% acetonitrile; 3~6 min, 60~95% acetonitrile; and 6~6.2 min, 10% acetonitrile. The flow rate was 0.6 mL·min^−1^. The column temperature was 40°C, and the sample plate temperature was 4°C. A sample volume of 10 μL was used, and the analysis time was 7 min.

Phytohormone analysis conditions were based on previous research and validated according to the published protocol (Pan et al., 2010) (Supplementary Table S5). The precision (relative standard deviation [RSD] <5%) and accuracy (recovery was from 82 to 114%) of this method met the requirements for quantitative determination (Supplementary Table S6). Linearity was verified using coefficients of determination (R^2^), which were all >0.99 within the adopted linear range (Supplementary Table S7).

For ginsenoside determinations, the same Waters ACQUITYUPLCBEH C18 column (2.1 × 100 mm, 1.7 μm) was used. The mobile phase was 0.05% formic acid water (A) and 0.1% (v/v) formic acid acetonitrile (B) with the following gradient: 28–32% B (0–2 min), 32% B (2–8 min), 32–35% B (8–10 min), 35% B (10–10.5 min), 35–45% B (10.5–11 min), 45–28% B (11–11.5 min), and 28% B (11.5–12 min). The equilibration time and the flow rate were set at 4 min and 0.5 mL/min, respectively. The injection volume of the sample was 1 μL. The autosampler temperature was set to 4°C. The conditions for determining ginsenosides were also based on previous research (Sun et al., 2016).

For the two chemical determinations, an ionization mode for ESI was used in the negative ion scan mode, the monitoring method was set for multiple reaction monitoring, the air curtain gas pressure was set at 30 psi, the ionization voltage was set at −4,500, the ion source temperature was set at 550°C, a spray gas pressure of 50 psi was used, and the auxiliary heating pressure was set at 50 psi. MultiQuant software was used to process the data.

## Results

### The Whole-Transcriptome Data Set Is Valuable for Dissecting Expression Profiles in Different Tissue Regions of Ginseng

To understand the phytohormone metabolism processes in different tissues, we analyzed global expression differences and similarities among the seven tissues using principal component (PC) analyses (Supplementary Figure S1). Different tissues from root to stem to leaf and roots from inner to outer were separated in the PC1. Further, different parts of the roots and leaves were separated along the PC2. These two PCs indicated that differences in the expression profiles could represent tissue differences.

### Ginsenoside Biosynthesis Is Significantly Enriched in the Leaf and Rhizome of Ginseng

Ginsenoside, a sort of triterpene saponin, is a major pharmacological ingredient in *P. ginseng*, and the biosynthetic enzymes and mode of regulation remain largely unknown. However, ginsenosides are mainly synthesized via the mevalonate (MVA) pathway in the cytosol. Further, 3-hydroxy-3-methylglutaryl CoA reductase (HMGR) is known as a rate-limiting enzyme in the mevalonate isoprene pathway in plants (Kim et al., 2014a). Ginseng contains two genes for HMGR: *HMGR1* and *HMGR2*. *HMGR1* is found abundantly in the rhizome, fiber root, and leaf blade of ginseng, while *HMGR2* is primarily expressed in the rhizome (Figure 1B). Previous studies have shown that *HMGR1* plays a general role in secondary metabolite production, whereas *HMGR2* is responsible for age-dependent ginsenoside accumulation in the roots (Kim et al., 2014a). Our findings further show that the leaf and rhizome are key tissues for the function of HMGRs in ginseng.

**Figure 2:**
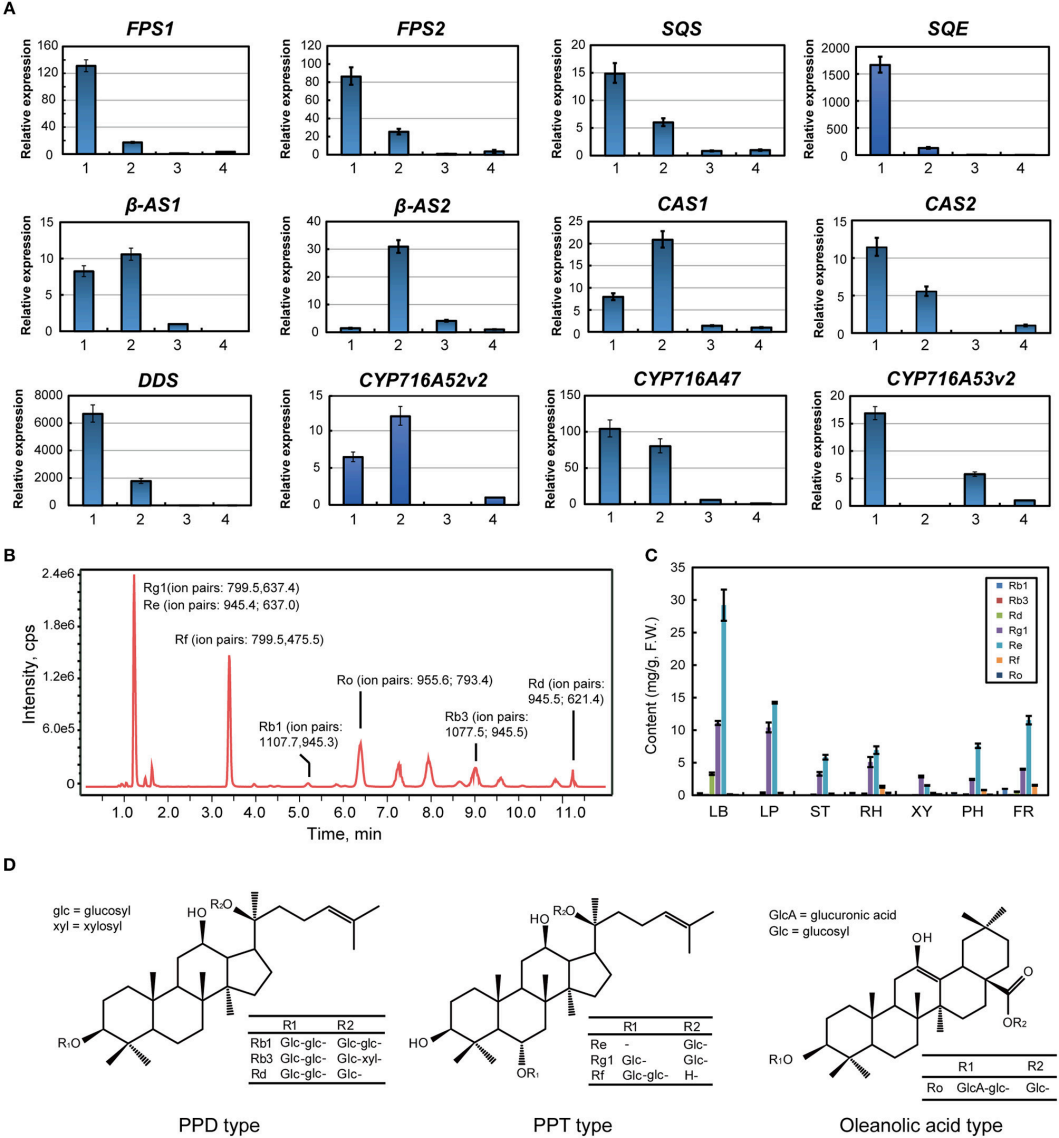
Analysis of ginsenoside synthesis and distribution in *Panax ginseng*. **(A)** Gene expression in the ginsenoside biosynthesis pathway via real time PCR. Red labeled unigenes were used for the primers designed in qPCR (Supplementary Table S1). Results from the 1. Leaf blade and leaf peduncle; 2. stem and rhizome; 3. xylem in the root; and 4. phloem and periderm in the root are shown. **(B)** Total ion ginsenoside chromatograms are shown. **(C)** Ginsenoside content in different ginseng tissues is shown. **(D)** Chemical structural formulas of the detected ginsenosides are shown.

Farnesyl diphosphate synthase (FPS) combines two molecules of isopentenyl diphosphate and one molecule of its isomer, dimethylallyl diphosphate, to farnesyl diphosphate; overexpression of *FPS* caused an ~2.4-fold increase in ginsenoside content in transgenic ginseng hairy roots and upregulated ginsenoside synthesizing genes in *P. ginseng* (Kim et al., 2014b). Our results show that FPS is mainly expressed in the leaf blade of ginseng, which is also validated by the qPCR data (Figures 1B, 2A). Squalene synthase (SQS) is a membrane-bound enzyme that condenses two farnesyl diphosphate (C15) molecules to yield C30-squalene. Squalene epoxidase(SQE), which catalyzes the oxygenation of the double bond of squalene to produce 2,3-oxidosqualene, represents a rate-limiting step in the phytosterol and triterpenoid saponin biosynthetic pathway (Kim et al., 2011). The accumulation of both SQS and SQE transcripts in the rhizome suggested that the rhizome is the key tissue to promote biosynthesis of 2,3-oxidosqualene, a ginsenoside precursor (Figures 1B, 2A).

2,3-Oxidosqualene is cyclized to produce dammarenediol, β-amyrin, and cycloartenol via dammarenediol synthase (DDS), β-amyrin synthase (β-AS), and cycloartenol synthase (CAS), respectively. Most β-AS and CAS genes were highly expressed in the underground tissues of ginseng, while DDS was mainly expressed in the leaf blade (Figures 1B, 2A). Three CYP genes involved in ginsenoside biosynthesis have been previously reported. CYP716A47 acts as a PPD synthase, yielding PPD. CYP716A53v2 catalyzes the generation of PPT from PPD. CYP716A52v2 modifies β-amyrin into oleanolic acid for the synthesis of an oleanane-type saponin. CYP716A47 and CYP716A52v2 transcripts were significantly enriched in the rhizome of ginseng, while CYP716A53v2 was highly expressed in the stem or xylem of ginseng (Figures 1B, 2A). Further, we investigated the distribution of PPD, PPT, and oleanolic acid (Figures 2B–D). Most PPD and PPT ginsenosides, such as Rd, Rg1, and Re, mainly accumulated in the leaf, while oleanolic acid, a Ro ginsenoside, was highly enriched in the rhizome.

Notably, there are numerous genes involved in ginsenoside biosynthesis that were significantly enriched in the leaf and rhizome of *P. ginseng*. In addition, both the transcriptome and chemical analyses suggested that PPD and PPT ginsenosides were largely synthesized in the leaf, and oleanolic acid was synthesized and distributed in the rhizome.

### Tissue Specificity of Endogenous Hormone Distribution in Ginseng

**Figure 3:**
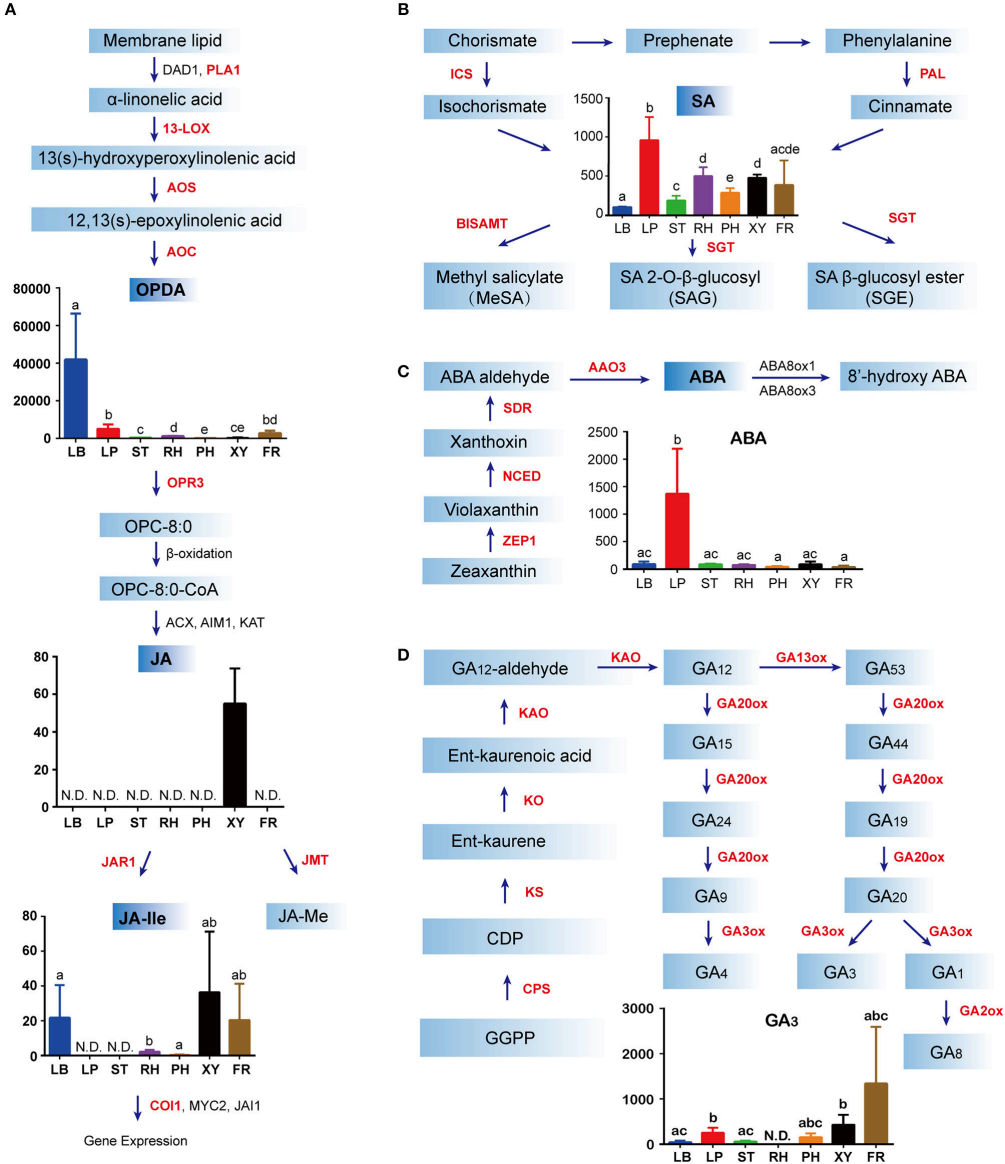
Biosynthesis and distribution of phytohormones in *Panax ginseng*. Jasmonates (JAs) **(A)**, abscisic acid (ABA) **(B)**, salicylic acid (SA) **(C)**, and gibberellins **(D)** in specific spatial regions are quantified as ng·g^−1^ fresh weight (FW) on the y-axis of each graph. For each tissue, the number of biological replicates was *n* = 3 unless stated otherwise. N.D. indicates not detected. Error bars indicate the standard deviation (SD). Significant differences in metabolite concentrations among the tissues were analyzed via analysis of variance and Tukey's multiple comparison test. Different letters indicate significant differences of the means (*P* < 0.05). Means with the same letter or with a common letter are not significantly different from each other. Red font indicates hormone metabolism genes detected in the RNA-Seq data. The colors in **(A–D)** indicate different regions of ginseng: blue, leaf blade (LB); red, leaf peduncle (LP); green, stem (ST); purple, rhizome (RH); orange, phloem and periderm in the main root (PH); black, xylem in the main root (XY); brown, fibrous root (FR). Enzyme abbreviations: AAO3, abscisic aldehyde oxidase 3; ABA8ox1 abscisic acid 8- hydroxylase 1; ABA8ox3, abscisic acid 8-hydroxylase 3; ACX, acyl-coA oxidase 3; AIM1, abnormal inflorescence meristem; AOC, allene oxide cyclase; AOS, allene oxide synthase; BISAMTL, benzothiadiazole salicylic acid carboxyl methyltransferases; COI, coronatine insensitive; CPS, copalyl diphosphate synthase; DAD1, defective in anther dehiscence 1; GA2OX1, gibberellin 2 oxidase 1; GA2OX8, gibberellin 2 oxidase 8; GA20OX3, gibberellin 20 oxidase 3; GA3ox1, gibberellin 3-beta-dioxygenase; ICS, isochorismate synthase; JAI1, jasmonate insensitive 1; JAR1, jasmonate resistant 1; JMT, jasmonic acid carboxyl methyltransferase; KAO, ent-kaurenoic acid oxidase; KAT, 3-keto-acyl-coA thiolase 1; NCED, 9-cisepoxycarotenoid dioxygenase; KO, ent-kaurene oxidase; KS, ent-kaurene synthase; MYC2, myelocytomatosis proteins 2; OPDA, (9S,13S)-12-oxo-phytodienoic acid; OPR3, oxophytodienoate reductase 3; PAL, phenylalanine ammonia lyase; PLA1, preferring phospholipase A1; SGT, sterol glucosyltransferase; SDR, short-chain dehydrogenase reductase; ZEP, zeaxanthin epoxidase; 13-LOX, 13-lipoxygenase.

Endogenous concentrations of phytohormones in different ginseng tissues, including JA, SA, GA, and ABA were quantified as previously described (Supplementary Figure S2). JAs are lipid-derived signals that mediate plant responses to biotic and abiotic stresses and in-plant developments. JA biosynthesis begins with α-linolenic acid, which is found in membrane lipids. α-Linolenic acid is a fatty acid that is converted to 13(s)-hydroperoxylinolenic acid by 13-lipoxygenase (13-LOX). After catalysis via allene oxide synthase (AOS) and allene oxide cyclase (AOC), 13(s)-hydroperoxylinolenic acid results in the formation of oxo-phytodienoic acid (OPDA), the highest concentration of which was found in the leaf blade, followed by the leaf peduncle (Figure 3A). Following reduction and three steps of β-oxidation, JA is formed. Interestingly, JA could only be detected in xylem from the ginseng main root, suggesting that the precursor of JA might be synthesized in the ginseng leaf and then transported to the other tissues of ginseng (Figure 3A). In addition, JA is converted to many other possible derivatives, including JA-Ile and JA-methionine. Accordingly, jasmonate resistant 1 and jasmonic acid carboxyl methyltransferase were shown to form JA-Ile and JA-methionine, respectively. JA-Ile was mainly detected in the leaf blade, in the xylem from the main root, and in the fibrous root, all of which were directly facing the environmental stressor (Figure 3A).

SA biosynthesis occurs via two pathways, including the isochorismate (ICS) and phenylalanine ammonia-lyase (PAL) pathways and is synthesized from chorismate by ICS and phenylalanine by PAL. Further, SA is then activated via glycosylation modification, methylation, or amino acid conjugation, which are increasingly recognized as integral to the spatial and temporal control of plant regulatory processes (Loake and Grant, 2007). SA was widely distributed in different parts of ginseng; however, it was predominantly found in the leaf peduncle region (Figure 3B), as determined via the chemical analysis results. Similarly, the highest ABA content was found in the leaf peduncle (Figure 3C). The first step of this specific ABA synthetic pathway is the two-step epoxidation of zeaxanthin to form all-trans-violaxanthin, which is catalyzed by zeaxanthin epoxidase (ZEP1). 9-Cis-epoxycarotenoid dioxygenase (NCED) catalyzed the oxidative cleavage of a 9-cis isomer of epoxycarotenoid to form xanthoxin. After the cleavage of 9-cis-epoxycarotenoids, xanthoxin is converted to ABA by short-chain dehydrogenase/reductase (SDR) and abscisic aldehyde oxidase 3 (AAO3) (Seo and Koshiba, 2002). Additionally, GA_3_ production was mainly detected in the leaf peduncle, in the xylem from the main root, and in the fibrous root of ginseng (Figure 3D). To initiate GA biosynthesis, GGDP is converted to ent-kaurene by two terpene cyclases: copalyl diphosphate synthase (CPS) and ent-kaurene synthase (KS). Ent-kaurene is then oxidized by ent-kaurenoic acid oxidase (KAO) to form GA_12_-aldehyde. In the final stage of this pathway, 2-oxo-glutarate dependent dioxygenases convert GA_12_-aldehyde to bioactive GAs, including GA 7-oxidase, GA 20-oxidase, GA 3β-dioxygenase, and GA 2-oxidase (Yamaguchi and Kamiya, 2000).

Taken together, these results suggest that GA is mainly distributed in fibrous roots, while stress phytohormones are mainly distributed on the ground. Furthermore, the leaf blade is an active site of JA biosynthesis, and ABA and SA are primarily found in the leaf peduncle.

### Hormone Levels Correlate With the Abundance of Hormone Biosynthesis Transcripts in Ginseng

**Figure 4:**
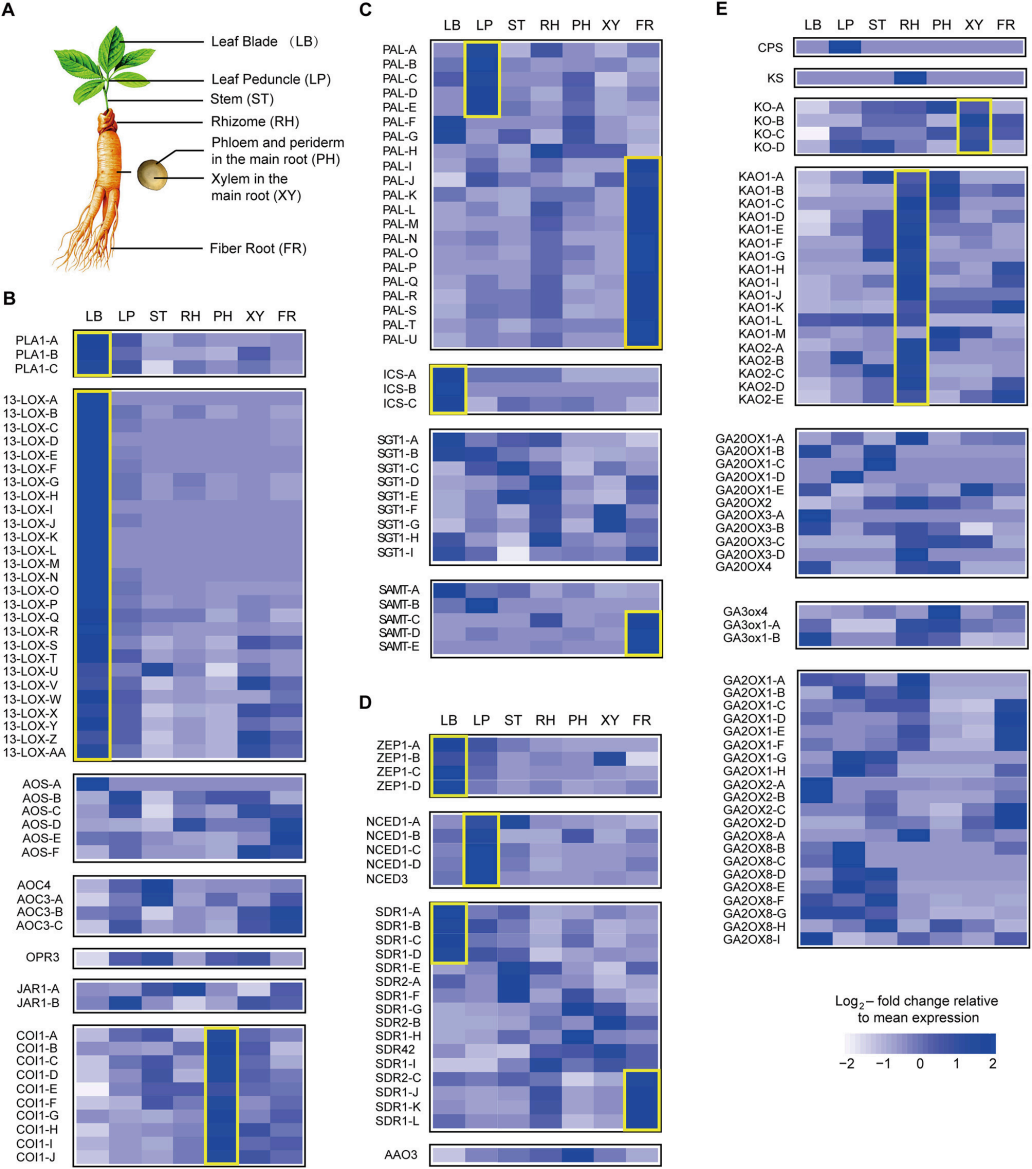
Expression pattern of hormone metabolism-related genes. Relative expression of selected genes responsible for key steps in the metabolism of seven plant hormones is shown using heatmap representations. The different tissues of ginseng are shown in **(A)**. Panels show jasmonate (JA) **(B)**, salicylic acid (SA) **(C)**, abscisic acid (ABA) **(D)**, and gibberellin (GA) **(E)** metabolism. Each column represents one tissue (LB, leaf blade; LP, leaf peduncle; ST, stem; RH, rhizome; XY, xylem in the main root; PH, phloem and periderm in the main root; FR, fibrous root) and each row represents one unigene corresponding in RNA-seq data (Supplementary Table S2). Different unigene labels might match to one annotated gene here due to the same specific sequence regions or domains. Colors show Z-score transformed gene expression values among all samples. Genes are grouped according to gene families or metabolic functions. Genes mentioned in the manuscript are highlighted with yellow boxes.

**Figure 5:**
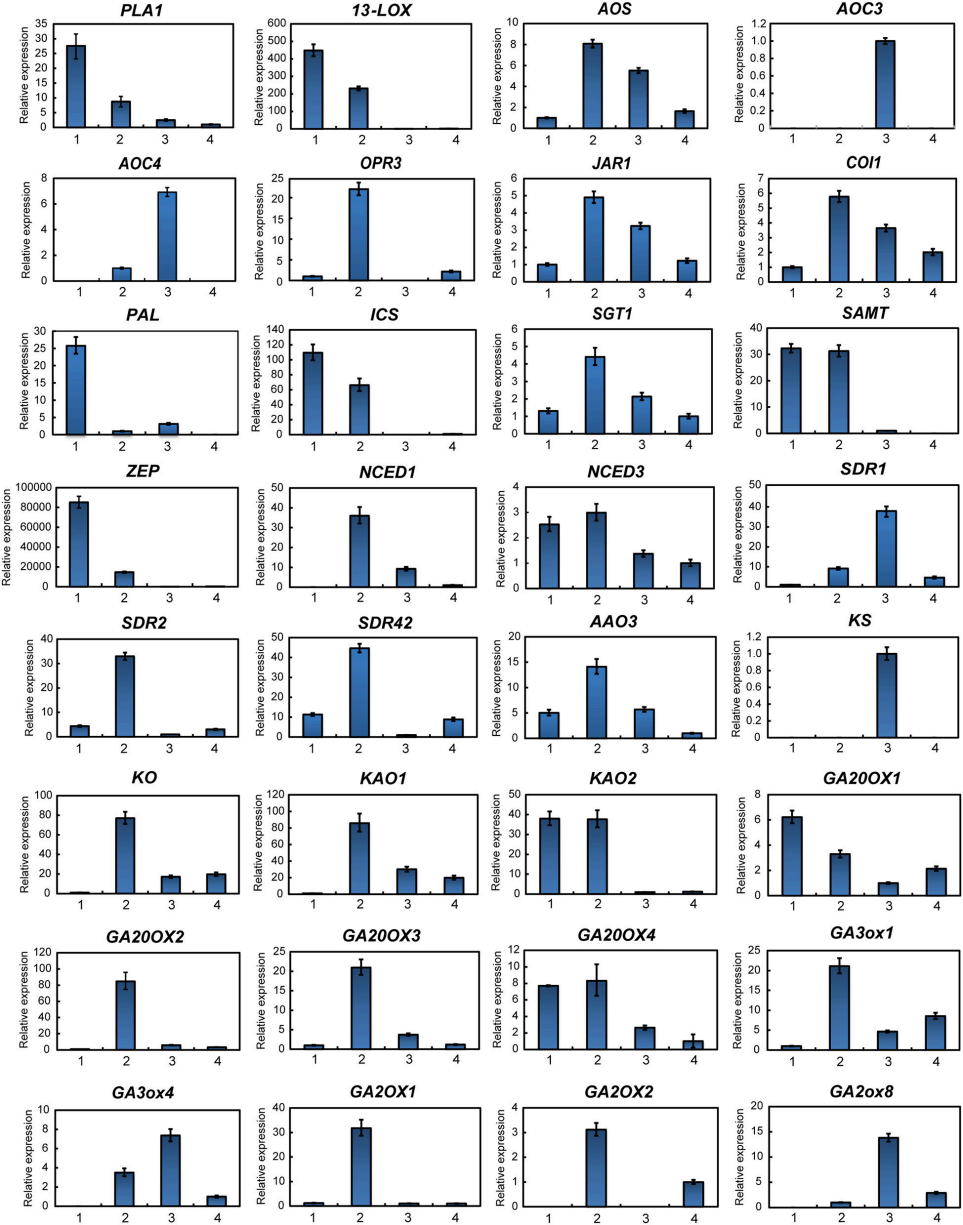
Gene expression of hormone metabolism-related genes detected via qPCR. Red labeled unigenes were used for the primers designed in qPCR (Supplementary Table S2). Results from the 1. Leaf blade and leaf peduncle; 2. stem and rhizome; 3. xylem in root; and 4. phloem and periderm in root are shown.

To further investigate hormone biosynthesis, we analyzed the expression of genes in different tissues of ginseng (Figure 4A). Increased expression of *PAL1* and *13-LOX*, which are involved in the early steps of JA biosynthesis, was observed in the leaf, consistent with the observed OPDA content (Figures 4B, 5). Further, *AOS* and *AOC*, genes associated with the two succeeding committed steps, were primarily expressed in the fibrous root and xylem of the main root (Figure 4B). The OPDA, JA, and JA-Ile levels reflected the expression pattern of JA biosynthetic genes (Figures 4A,B). However, JA was undetected in most tissues, except for the xylem. This is likely attributable to JA being converted to other derivatives such as JA-Ile and JA-methionine. Coronatine insensitive 1 (COI1) interacts with jasmonate zim (JAZ) proteins and targets them for degradation in response to JA signaling, suggesting that COI1 is highly expressed and has a strong JA signaling response. Interestingly, COI1 was highly expressed in the underground ginseng tissues, suggesting that the rhizome and root might face more stress than other tissues (Figures 4B, 5).

SA, a plant defense hormone, is primarily synthesized via the ICS route during plant immunity processes (Wildermuth et al., 2001). Genes associated with the PAL pathway were found to be highly expressed in the leaf peduncle and fibrous root during the transcriptomic analyses (Figures 4C, 5), suggesting that the PAL pathway greatly contributes to SA production. Simultaneously, genes associated with the ICS pathway were primary expressed in the leaf blade. Differences in SA content between the leaf blade and leaf peduncle tissues may be explained by the expression of sterol glucosyltransferase genes (Figures 4C, 5), catalyzing the formation of both SA and 2-O-β-d-glucoside, as well as the glucose ester of SA and possibly regulating SA levels in the leaf blade.

We further analyzed expression of zeaxanthin epoxidase (*ZEP*) and 9-cisepoxycarotenoid dioxygenase (*NCED*), abscisic acid (ABA) biosynthetic genes. The gene expression patterns associated with NCED biosynthesis in the leaf peduncle support the results showing significantly more accumulation of ABA in the leaf peduncle than in other tissues (Figures 3C, 4D, 5).

Among all seven analyzed tissues, the leaf peduncle, rhizome, and fibrous root regions showed the highest concentration of GAs (Figures 4E, 5). Ent-copalyl diphosphate synthase and gibberellin 2 oxidase 8 were highly expressed in the leaf peduncle; ent-kaurene synthase, ent-kaurenoic acid oxidase, gibberellin 20 oxidase 3, gibberellin 3-beta-dioxygenase, and gibberellin 2 oxidase 1 were mainly expressed in the rhizome; ent-kaurene oxidase was highly expressed in xylem from the ginseng main root; and gibberellin 20 oxidase 1 was also highly expressed in the fibrous root of ginseng (Figures 4E, 5).

## Discussion

The demand for ginsenosides is increasing, attributable to the recognition of a variety of pharmacological activities and the development of related ginsenoside products. Thus, it is crucial to elucidate the highly intrinsic relationship in endogenous phytohormones and ginsenosides. To date, several reports have suggested that rate-limiting enzymes of the mevalonate isoprene and cytochrome P450 (CYP) pathways are involved in ginsenoside biosynthesis (Han et al., 2011; Kim et al., 2014b). However, the correlation mechanisms underlying hormone distribution and ginsenoside biosynthesis are largely unknown. In this study, hormone profiling and expression of hormone biosynthetic genes indicate that tissue-specific accumulation of ginsenosides is closely related to phytohormones. A correlation analysis revealed that most genes involved in ginsenoside biosynthesis are related to each other (Supplementary Figure S3), suggesting that these genes might be regulated by similar upstream phytohormones.

Several studies have shown that JA plays an important role in plant-insect interactions and defense responses against pathogen invasion (Liechti and Farmer, 2003). Ginsenosides, as defensive secondary metabolites, are effectively stimulated by JA (Wang et al., 2005). For instance, JA responsive transcription factors, including apetala2/ethylene response factors (AP2/ERFs), myelobastosis (MYBs), and WRKYs could be involved in ginsenoside biosynthesis (De Geyter et al., 2012; Subramaniyam et al., 2014). In our study, total ginsenoside content was highest in the leaves among the seven tissues examined (Figure 2C). Key synthetic genes in ginsenoside biosynthesis, including *HMGR, FPS*, and *DDS*, which are triggered by JA (Lee et al., 2004; Kim et al., 2007, 2009; Wang et al., 2013), are also mainly expressed in the leaf. Corresponding to this phenomenon, of the four major endogenous hormones studied, only OPDA and JA active substance JA-Ile (both JA precursors) significantly accumulated in the leaf. These data suggest that dammarenediol-type ginsenosides are first formed in the ginseng leaf and are related to the JA-signal pathway. Therefore, *PLA* and *LOX*, which mainly appear in the leaf, may make major contributions to the accumulation of total ginsenosides in the leaf. *PgLOX6*, which encodes a lipoxygenase, contributes to JA biosynthesis and ginsenoside production in *P. ginseng* and provides a molecular tool for increasing ginsenoside production (Rahimi et al., 2016). However, the expression of JA-related genes in the xylem remains low, indicating that the xylem should be a component of the JA transport system, but is not central to JA biosynthesis. These results are similar to those obtained through research of *Arabidopsis thaliana* plant defense responses to wounding and herbivore attack, which are regulated by signal transduction pathways that operate both at the site of wounding and in undamaged distal locations via JA signaling (Li et al., 2002). JA-Ile, which is the most biologically active compound and the ligand for the Skp, Cullin, F-box containing complex-jasmonate zim (SCF-COI1-JAZ) co-receptor complex, mainly appears in the leaf blade and fiber root. The distribution of JA-Ile and ginsenosides corresponds with the natural growth state of plants and demonstrates that the leaf and fiber root also play an important role in plant defenses.

SA is a plant signaling molecule involved in some signal transduction pathways in the defense response that induce enzymes of the secondary metabolic pathway (Hahlbrock et al., 2003). SA is synthesized from chorismate in plastids via two routes and is a product of the shikimate pathway. One route is via ICS, which is responsible for >90% of SA synthesized during activation of the stress response (Wildermuth et al., 2001). The other route primarily involves SA synthesis in the absence of stress and is a PAL-mediated pathway. There were no significant tissue-specific differences in the level of SA, which is inconsistent with the high expression of SA biosynthesis in the leaf and fibrous root. This phenomenon may be attributable to cross talk between SA and JA, which decreases the SA content level in the leaf and fibrous root. In most plant physiological processes, signaling crosstalk between SA and JA commonly manifests as a reciprocal antagonism and may be adaptive (Thaler et al., 2012). This is also consistent with previous studies in which JA was shown to be a strong effective elicitor to stimulate the biosynthesis of ginsenosides in all *P. ginseng* cell lines (Kim et al., 2007, 2009). This may imply that JA plays an essential role in the process for endogenous plant hormones participating in the regulation of ginsenoside biosynthesis. However, the concentration of ginsenosides is higher in the petiole than would be expected based on the low expression of synthetic genes in this tissue. Further, abscisic acid (ABA) appears to be enriched in the petiole, which may regulate the transport of ginsenosides and the location of this enrichment. Particularly, *NCED* plays an indispensable role during this process. Both our profiling data and qPCR analyses further support the above conclusion and suggest that the leaf blade and leaf peduncle are important during the defense response. Co-localization and the synergistic activity of the stress hormones JA, SA, and ABA in the different tissues of ginseng may indicate their role in mediating the expression of genes related to ginsenoside biosynthesis.

GA_3_ might also influence ginsenoside content. Expression of ent-kaurene oxidase and ent-kaurenoic acid oxidase in the fibrous root and xylem, respectively, may explain the accumulation of GAs in the root region, indicating a role in growth regulation of newly emerging tissues and lateral organs. It is also likely that GAs may regulate the process in an auxin-dependent manner, attributable to auxin having a major role in almost all steps of fibrous root initiation and development (Gou et al., 2010). Additionally, the transcriptomic data between ginsenoside synthesis and GA biosynthesis reported in this study suggest that endogenous GA may play a positive role in the regulation of ginsenoside synthesis.

In this study, we evaluated the coordinated expression of hormone biosynthesis genes along with hormone concentrations, indicating the potential regulation of medical secondary metabolism in ginseng (Supplementary Figure S4). High ginsenoside levels appear in the leaf, petiole, and fibrous root, where the four main hormones also match the expression of ginsenoside synthetic genes. This overlap indicates an intrinsic high relevance between endogenous phytohormones and ginsenoside synthetic genes. Our data presented here will be useful for further studies regarding the function of genes involved in the regulation of ginsenoside biosynthesis.

## Data Availability Statement

The seven RNA-seq library datasets (leaf blade, leaf peduncle, stem, rhizome, phloem and periderm in the main root, xylem in the main root, and fiber root) for this study can be found in the National Center for Biotechnology Information (NCBI) Sequence Read Archive (SRA: PRJNA302556).

## Author Contributions

YY and LH coordinated the project, directed the work, and revised the manuscript. KC and JL analyzed and wrote the manuscript. KC, RJ, and XZ conceived and conducted the plant hormone analyses using ultra-performance liquid chromatography—tandem mass spectrometry (UPLC-MS). KC, RJ, and JY conducted the ginsenoside analyses using ultra-performance liquid chromatography—tandem mass spectrometry (UPLC-MS). TC assembled the transcriptomic ginseng data. TC and JL analyzed the gene expression data involved in ginsenoside and phytohormone biosynthesis. RJ, YT, and CJ collected the experimental samples. JZ, YZ, and YJ cultured the ginseng seedlings.

### Conflict of Interest Statement

The authors declare that the research was conducted in the absence of any commercial or financial relationships that could be construed as a potential conflict of interest.
