# Supplementary material for: Biogenic Synthesis and Spatial Distribution of Endogenous Phytohormones and Ginsenosides Provide Insights on Their Intrinsic Relevance in Panax ginseng
Source: Front Plant Sci. 2019 Jan 9;9:1951. doi: 10.3389/fpls.2018.01951 (PMC6333697; doi:10.3389/fpls.2018.01951)
Supplement: Supplementary file 1 [file Table_1.DOCX]

Supplemental Figures


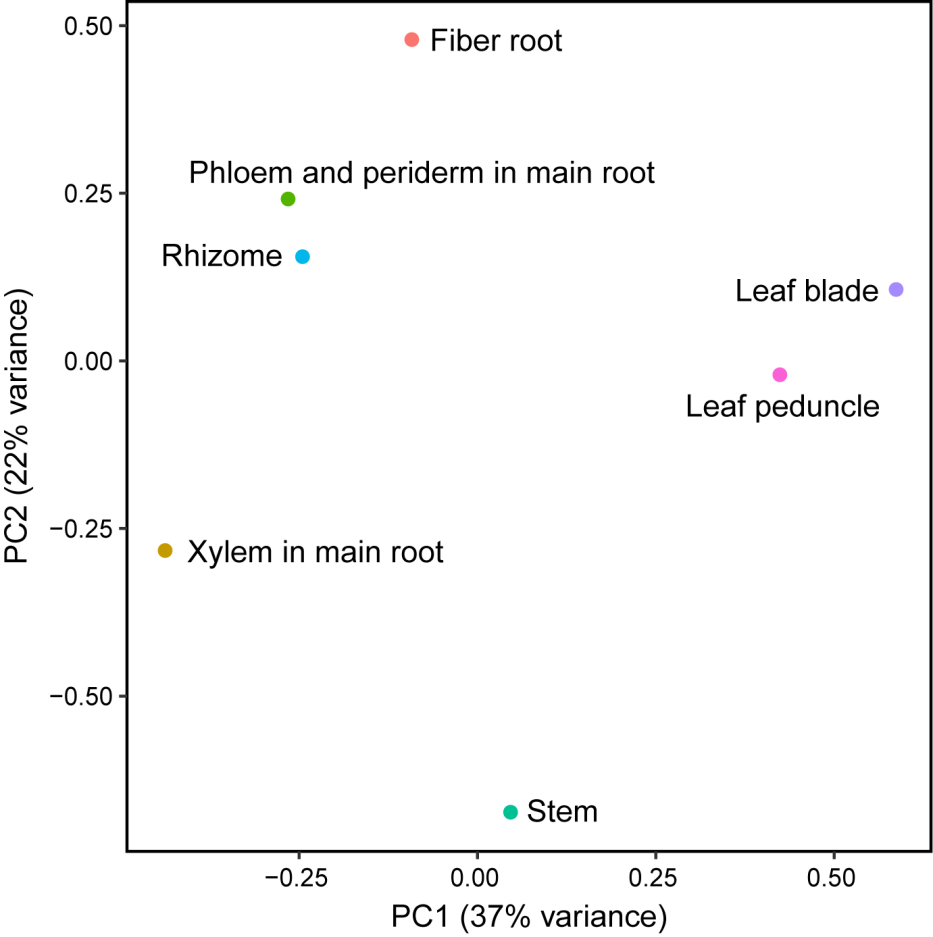


**Supplemental Fig. S1** Global expression differences and similarities among the seven tissues using principal component analysis (PCA).


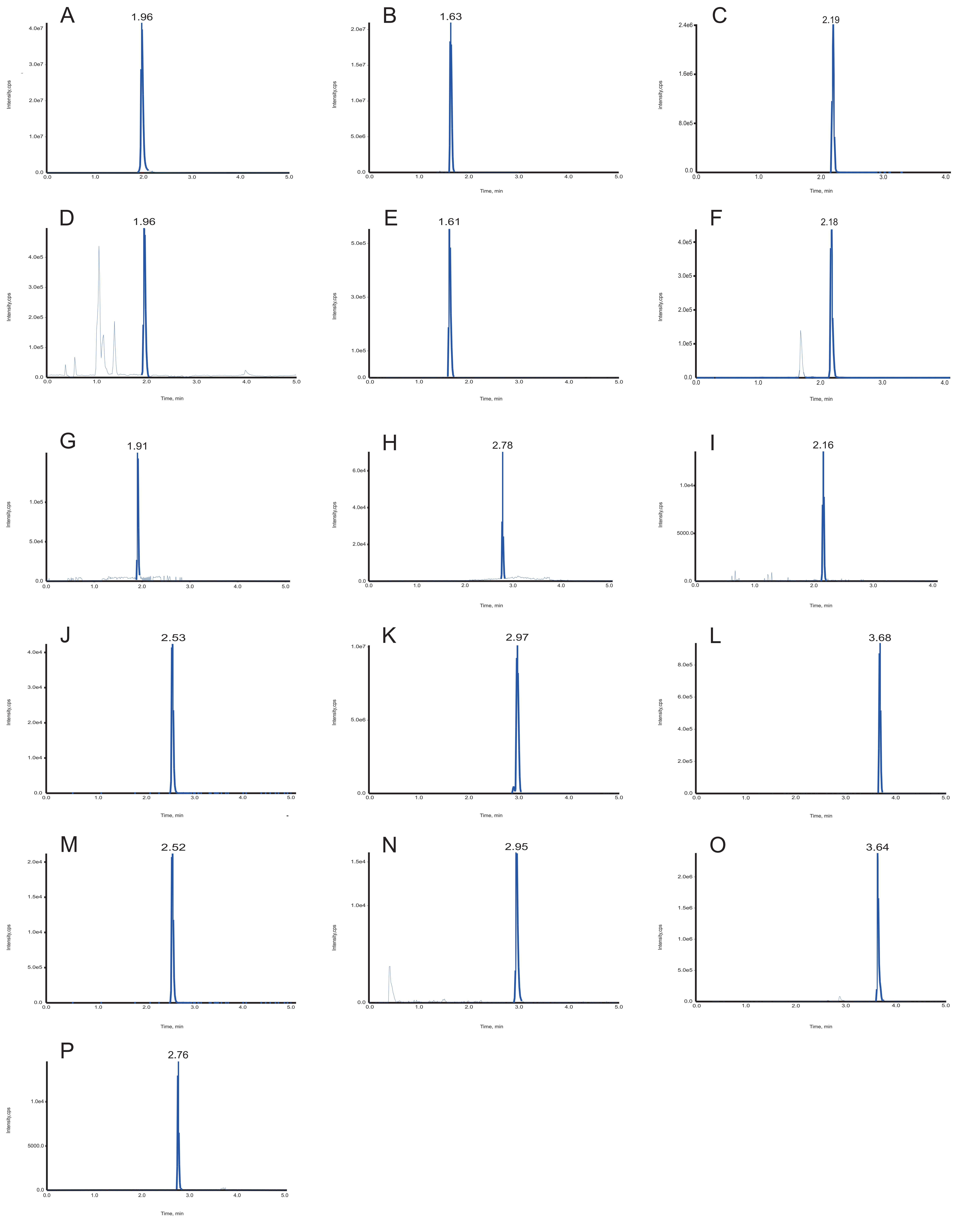


**Supplemental Fig. S2** Selective reaction monitoring ion flow chart. A: SA standard; B: GA_3_ standard；C: ABA standard; D: SA sample; E: GA_3_ sample; F: ABA sample; G: ^2^H_4_-SA standard; H: ^2^H_2_-GA_4_ standard; I: ^2^H_6_-ABA standard; J: JA standard; K: JA-Ile standard; L: OPDA standard; M: JA sample; N: JA-Ile sample; O: OPDA sample; P: ^2^H_2_-JA standard.

**
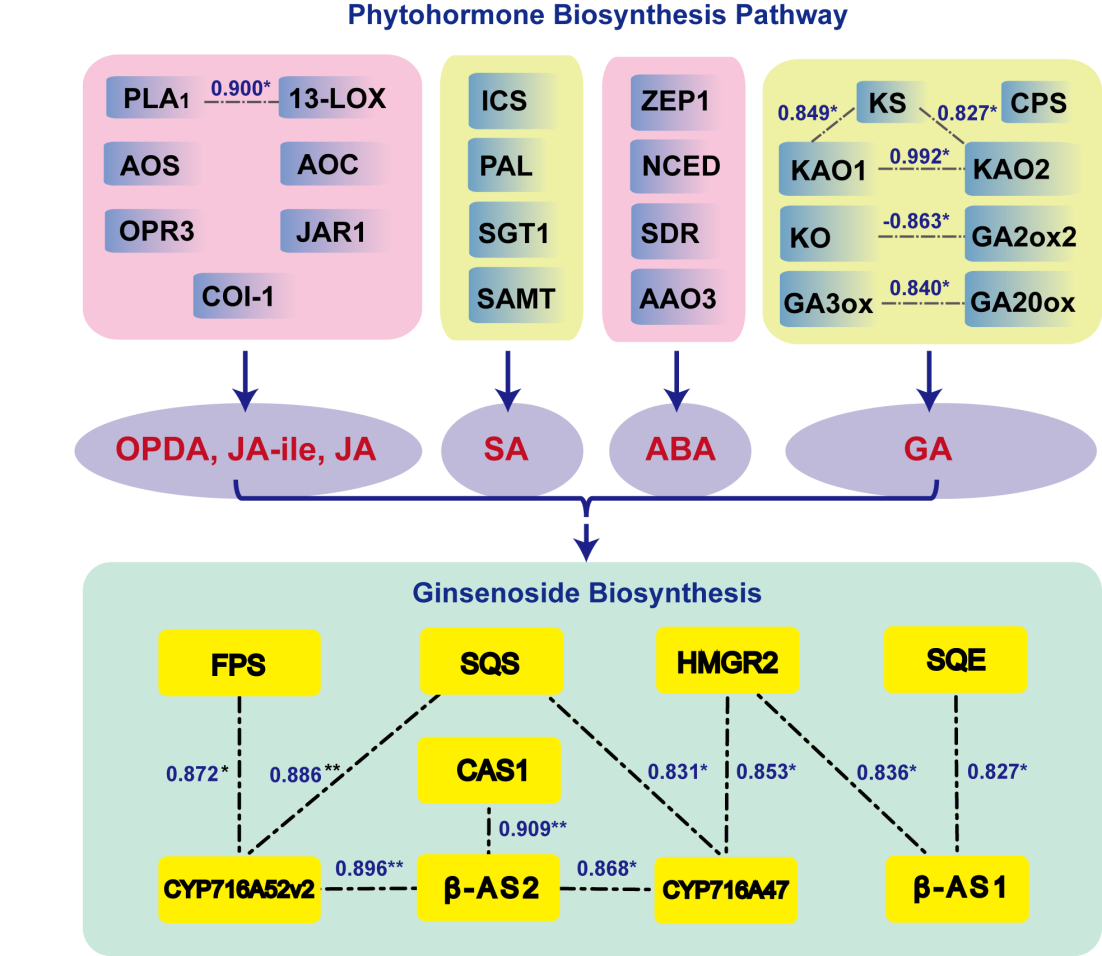
**

**Supplemental Fig. S3** Correlation analysis of genes involved in the biosynthesis of jasmonates (JA), salicylic acid (SA), abscisic acid (ABA), gibberellin (GA), and ginsenoside. A correlation coefficient > 0.8 demonstrates a highly-positive correlation. **P* < 0.05. ***P* < 0.01.

**
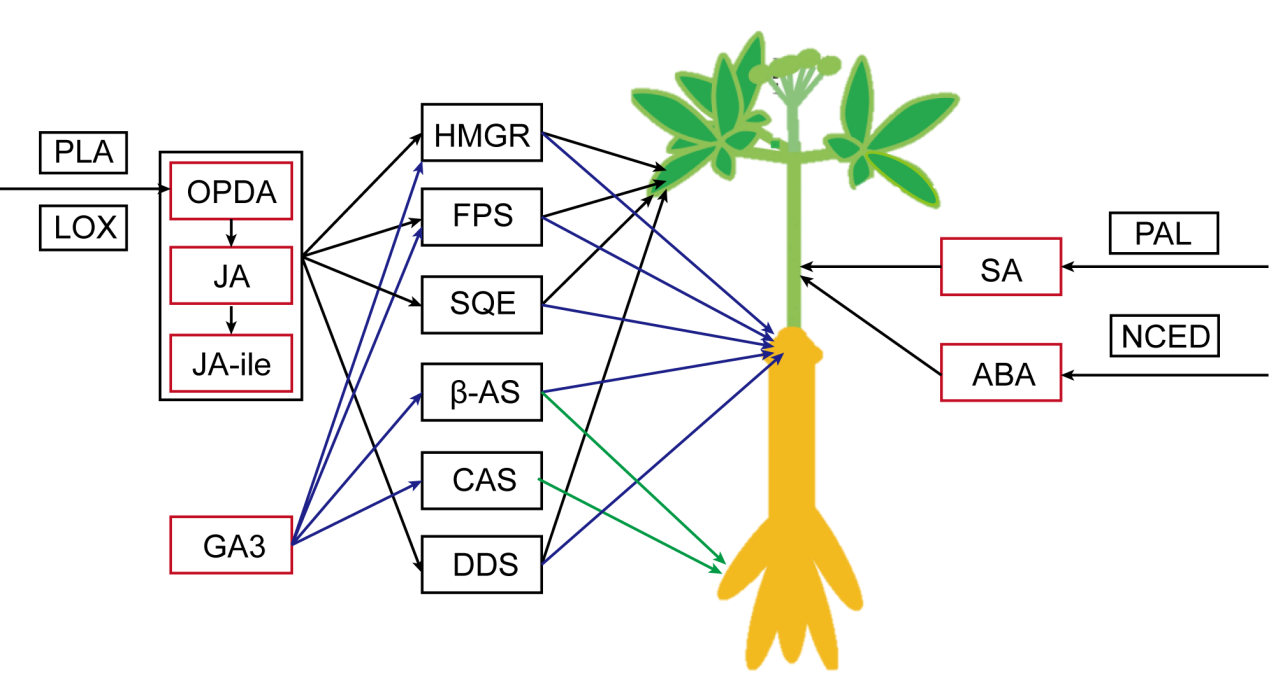
**

**Supplemental Fig. S4** Hormone and ginsenoside biosynthetic gene expression profiling. Red boxes include hormone names; black boxes include biosynthetic gene names.
